# Socioeconomic Inequalities in the Prevalence of Diabetes in Argentina: A Repeated Cross-Sectional Study in Urban Women and Men

**DOI:** 10.3390/ijerph19158888

**Published:** 2022-07-22

**Authors:** Carlos Rojas-Roque, Akram Hernández-Vásquez, Diego Azañedo, Guido Bendezu-Quispe

**Affiliations:** 1 Health Technology Assessment and Health Economics Department, Institute for Clinical Effectiveness and Health Policy, Buenos Aires C1414CPV, Argentina; crojas@iecs.org.ar; 2 Centro de Excelencia en Investigaciones Económicas y Sociales en Salud, Vicerrectorado de Investigación, Universidad San Ignacio de Loyola, Lima 15024, Peru; 3 Facultad de Ciencias de la Salud, Universidad Científica del Sur, Lima 15067, Peru; dazanedo@cientifica.edu.pe; 4 Centro de Investigación Epidemiológica en Salud Global, Universidad Privada Norbert Wiener, Lima 15046, Peru; guidobq@gmail.com

**Keywords:** diabetes mellitus, healthcare disparities, epidemiology, Argentina

## Abstract

This study measured the socioeconomic inequalities in the prevalence of diabetes between 2005 and 2018 in an urban Argentinian population. Data were obtained from the repeated cross-sectional surveys “National Survey of Risk Factors” (ENFR is its acronym in Spanish). From 2005 to 2018, four rounds of ENFR were administered to men and women over 18 years of age. Concentration curves (CC) and the Erreygers concentration index (ECI) were used to describe the socioeconomic inequalities in diabetes’ prevalence. A decomposition analysis was performed to determine the contribution of each variable to inequality in diabetes’ prevalence. Data from 41,219 (2005), 34,583 (2009), 32,232 (2013), and 29,094 (2018) individuals were analyzed. Women reported a greater prevalence of diabetes compared with men for all the years included. According to the CC and ECI, we found no evidence of inequality in men throughout all study years. For women, throughout all years, the CCs were above the line of equity, and the ECIs during all the years were negative and different from zero (*p* < 0.01). For women, we found no evidence of a reduction in inequalities between 2005 and 2018 (*p* = 0.475). The socioeconomic inequality for women was largely driven by public insurance, primary and secondary education, and employment. Diabetes’ prevalence was not associated with socioeconomic status in men, while the prevalence of diabetes in women was more concentrated among poorer women. During the 13 years, there was no evidence of a reduction of inequality in women, noting that interventions must prioritize and should focus on the main contribution of inequalities, such as education and employment.

## 1. Introduction

Diabetes is a chronic progressive disease that has increased in both the number of cases and its prevalence worldwide, partly due to improvements in ability, technology, and awareness for diagnosing diabetes even in developing countries. According to the Global Report on Diabetes, the number of adults with diabetes increased from 108 million during 1980 to 422 million in 2014, and the prevalence increased from 4.7% to 8.5% in the same period [1]. The increase in the prevalence of the disease is faster in low- and middle-income countries [1]. Furthermore, there was a 5% increase in premature mortality attributed to diabetes between 2000 and 2016. In 2021, approximately 1.6 million deaths in the world were attributed to this disease [1]. The impact of diabetes on the global burden of disease is important since, between 2000 and 2019, diabetes has gone from being the fifteenth cause of death in the world to occupying the ninth position [2] as one of the leading causes of death [3].

The Latin America and the Caribbean (LAC) region is one of the world’s regions most affected by diabetes. In this region, the prevalence of the disease in people 20 to 79 years of age has increased from 7.44% in 2010 to 9.70% in 2019 [4]. In Argentina, the prevalence of diabetes is rising according to the pattern in LAC [5,6]. Although it is described that this increase could be attributed to an improvement in access to preventive care services [7], at the population level, approximately one in ten Argentines has diabetes, irrespective of sex, making this disease a public health problem for the country. Thus, diabetes represents a significant disease burden in Argentina as the cause of 3% of all deaths for all ages.

In high-income countries, socioeconomic inequalities have been described in the prevalence of chronic conditions, such as diabetes, showing a gradient in which the poorest have an increased probability of having both diabetes and complications related to this disease [8]. However, only a few studies have studied inequalities in diabetes’ prevalence at the national level in LAC, including Argentina, a country with marked socioeconomic differences in the prevalence of other chronic conditions such as hypertension [9]. In addition, little is known about how socioeconomic inequalities vary across time. Hence, the aim of this study was to measure the socioeconomic inequalities in diabetes among an urban population of adult men and women in Argentina between 2005 and 2018. This information will aid in the design of public health policies and strategies for reducing the burden of and early deaths by non-communicable chronic diseases.

## 2. Methods

### 2.1. Data Source

To estimate socioeconomic inequalities, data on diabetes’ prevalence and individual characteristics were obtained from the repeated cross-sectional survey “National Survey of Risk Factors” (ENFR is its acronym in Spanish). The survey contains nationally representative data on risk factors, health care utilization, and the prevalence of non-communicable diseases in Argentinians over the age of 18. From 2005 to 2018, four rounds of ENFR were administered by the National Institute of Statistic and Census (INDEC is its acronym in Spanish) and the Ministry of Health of Argentina. The ENFR sample design is probabilistic, stratified, multi-stage, and representative at the urban national and urban provincial levels. Further methodological details can be found in the final technical report [10].

### 2.2. Sample Size

**Figure 1:**
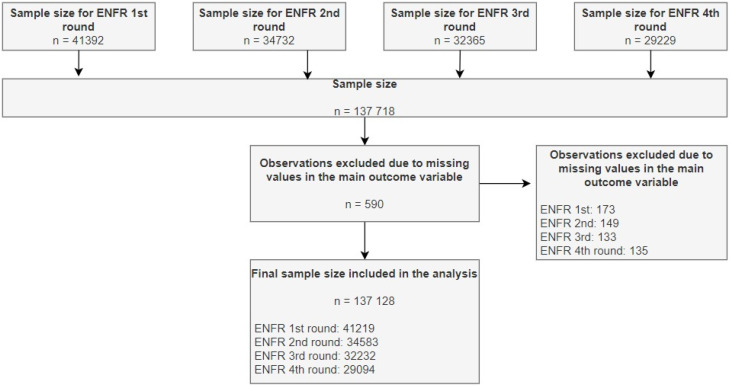
Study flowchart.

Eligible participants were defined as people 18 years or older who were residents of urban locations with 5000 or more inhabitants. The flowchart of the sample size of the study is depicted in Figure 1. The final sample size was composed of 41,392 individuals for the first round of ENFR and 34,732, 32,365, and 29,229 individuals for the second, third, and fourth rounds, respectively. However, for the analysis, 173, 149, 133, and 135 observations were excluded from the first, second, third, and fourth rounds of the ENFR, respectively, because they did not report the values of the main outcome variable. Therefore, the sample size included in the analysis was composed of 41,219, 34,583, 32,232, and 29,094 individuals in the first, second, third, and fourth rounds of the ENFR, respectively.

### 2.3. Outcome Variable

The outcome of interest was a dichotomous variable of diabetes (yes/no). Diabetes was considered when individuals were told in the past that they had diabetes by a health professional (physician, nurse); otherwise, diabetes was not considered. Diabetes data was collected by self-reporting. In the fourth round, the ENFR had both the self-report and clinical measurements of diabetes. According to the data of the survey, the self-report implies a low underestimation for chronic health conditions, for example, 4.5 percentage points (p.p.) for the case of overweight or obesity, 5.8 (p.p.) for hypertension, and 4.3 (p.p.) for diabetes. The proportion of diabetes detected by self-report in Argentina was similar to those reported in the previous studies [11,12]. Thus, we considered the self-report to be a quite reliable approach to measuring diabetes’ prevalence in Argentina. Our self-report approach to measure the inequalities in the prevalence of diabetes was in line with those of previous studies [13,14,15].

### 2.4. Independent Variables

The independent variable was the household income per-capita. The information on household income was collected in the questionnaire through two questions aimed at capturing the total monthly household income of the individual. The first question asked about the amount received at home in the past month, including income from work, retirement, rents, unemployment insurance, scholarships, food payments, or other sources of income. For individuals who did not know the amount or did not answer, the second question was applied to ask about the interval over which the total monthly income of the household was included. To estimate the missing values in the income variables, an imputation was applied with the hierarchical hot-deck method, with further details available in the final technical report of ENFR [10]. To adjust the income according to the household size, an equivalence scale equal to the square root of the number of household members was considered, using the consumer unit criteria of the Organization for Economic Cooperation and Development (OECD) [16].

In addition, to describe the population, we included the following variables in accordance with previous studies describing the variables that affect health [17] and based on studies that estimated inequality in the prevalence of diabetes [18,19,20]: sex (men/women), age in years (18–29/30–59/60 or more), married or cohabiting (yes/no), education (none/primary/secondary/higher), type of health insurance (private insurance/social security insurance/public insurance), and currently employed (yes/no).

### 2.5. Statistical Analysis

All the analyses were carried out using Stata 14.2 (StataCorp., College Station, TX, USA, 2016). Each analysis was weighted to account for the individual survey sample design, non-response, and the cluster size. For the analyses, we grouped the household income-per-capita by quintiles and performed separate analyses for men and women [21]. Weighted proportion together with its 95% confidence interval (95% CI) were used to describe the demographics and socioeconomic sample characteristics, as well as the prevalence of diabetes in men and women.

To describe the socioeconomic inequalities in diabetes prevalence, we used concentration curves (CC) and a concentration index. CC plot the cumulative percentage of diabetes (*y*-axis) against the cumulative percentage of the population (*x*-axis), ranked according to the household income per capita, starting with the poorest and ending with the wealthiest individuals. If the prevalence of diabetes was evenly distributed according to socioeconomic status (no inequality), the CC would be a 45-degree line plotted from the bottom left-hand corner to the upper right-hand corner. This line is known as the line of equity. By contrast, if diabetes’ prevalence assumes higher (lower) values among poorer individuals, the CC would then lie above (below) the line of equity. The farther the CC are above the line of equality, the more concentrated the diabetes is among the poor individuals, or the farther the CC are below the line of equity, the more concentrated the diabetes’ prevalence is among the wealthiest individuals [22,23].

The concentration index is a relative measure of inequality, defined as twice the area between the CC and the line of equity. Mathematically, it is defined as $\frac{2}{µ}cov\left(h,r\right)$, where μ represents the mean prevalence of diabetes, h represents the health variable (diabetes), and r is the cumulative percentage that each individual represents over the total population after ranking diabetes’ prevalence by the household income per capita [23]. The concentration index ranges from μ−1 and 1−µ [24]. If the CC lie above (below) the line of equity, the concentration index has a negative (positive) value. The higher the absolute value of the concentration index is, the higher the magnitude of socioeconomic inequality is. To consider the bounded nature of diabetes’ prevalence (yes/no), we performed the Erreygers concentration index (ECI) [25] using the *“conindex”* command in Stata [26]. ECI can be defined as ECI=4∗µ∗concentration index (y), where y is the health variable (diabetes).

To determine the contribution of each variable to the inequality in diabetes, we decomposed the ECI using generalized linear models (GLM). In comparison with other approaches such as the ordinary least squares (OLS) or Probit estimations, GLM was demonstrated to be the best choice when decomposing a binary variable [27]. We can express the model of diabetes (y) as follows:

(1) $$y=\alpha +\sum_{k}\beta_{k}X_{k}+\varepsilon$$

in which α is an intercept, $X_{k}$ represents a set of variables that predict y, $\beta_{k}$ is the coefficient of $X_{k}$, and ε is the stochastic term of error. Using this equation, ECI can be decomposed as follows:

(2) $$ECI=4\left[\overset{j}{\underset{j=1}{\sum }}\beta_{k}{\bar{X}}_{k}*CI+GCI_{\varepsilon }\right]$$

in which $\beta_{k}$ is the partial effect of diabetes, CI is the concentration index of $X_{k},$ and $GCI_{\varepsilon }$ is the generalized concentration index of the stochastic term of error. The equation suggests that a variable contributes to inequality in the prevalence of diabetes when it is correlated with diabetes’ prevalence and the variable is unequally distributed across the household income per capita quintile. The higher the partial effect of a variable is and the more unequally that variable is distributed with respect to household income per capita, the higher the contribution of that variable is to inequality [23].

Decomposition brings elasticity, concentration, contribution, and percentage of contribution to the inequality for each variable. Elasticity denotes the change in the dependent variable (diabetes) associated with a one-unit change in the independent variable. A positive or negative sign in elasticity indicates an increasing or decreasing probability of diabetes’ prevalence in association with a change in the independent variable, respectively [23]. The concentration index represents the concentration index of the variables with reference to the household income per capita quintile. A positive or negative value means that diabetes’ prevalence is more frequent among the wealthiest or poorest households, respectively. Lastly, the contribution and percentage contribution represent the absolute and relative contribution of each variable included in the model to the overall socioeconomic-related inequality in diabetes’ prevalence. A positive or negative contribution or percentage contribution in a variable result in an increase or decrease in the observed socioeconomic inequality, respectively [23].

### 2.6. Ethics Statement

This study did not require the approval of an institutional ethics committee since all the databases are fully anonymized and are freely and publicly available from the website of INDEC (https://www.indec.gob.ar/ accessed on 26 May 2022).

## 3. Results

Table 1 summarizes the characteristics of the study sample. Data from 41,219 (2005), 34,583 (2009), 32,232 (2013), and 29,094 (2018) respondents were analyzed. In all years, the sample comprised slightly more women than men (on average, 52% were females). The mean age of the individuals in all years was around 43 years, and approximately six out of ten respondents were married or cohabiting. Except for 2005, in all rounds, most of the individuals had a secondary education. Nearly half of individuals had Social Security insurance, while nearly one-third had public insurance. Six out of ten individuals were employed, and the majority were from a metropolitan area and the Pampeana geographical area of Argentina.

**Figure 2:**
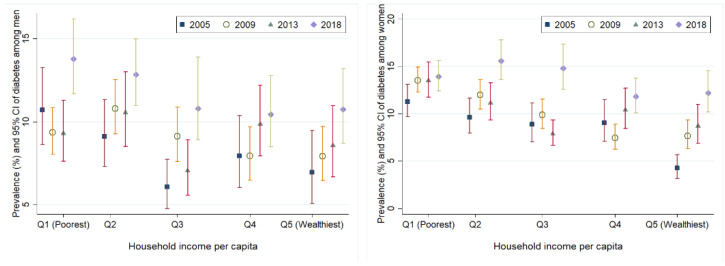
Prevalence of diabetes according to sex and household income per capita.

Women (8.83% (2005), 10.19% (2009), 10.39% (2013), and 13.69% (2018)) reported a greater prevalence of diabetes compared to men (8.04% (2005), 8.97% (2009), 9.09% (2013), and 11.70% (2018)). Figure 2 shows the prevalence of diabetes according to sex and household income per capita. In 2005, the prevalence of diabetes in men was 10.7% (95% CI: 8.64% to 13.27%) for the poorest individuals and 7% (95% CI: 5.09% to 9.47%) for the wealthiest individuals (difference of 3.7 percentage points, 95% CI: 0.65 to 6.88, *p* = 0.020). We did not find a gap in the prevalence of diabetes between the poorest and wealthiest during 2018 (13.8% vs. 10.7% respectively, difference of 3.1 percentage points, 95% CI: −0.01 to 6.21, *p* = 0.059). Over the 13 years, the prevalence of diabetes among the wealthiest individuals significantly increased by 3.7 percentage points (95% CI: 0.65 to 6.88, *p* = 0.018). However, among the poorest individuals, there was an increase in the prevalence of three percentage points, although this increase was not significant (95% CI: −0.15 to 6.27, *p* = 0.063). For women, in 2005, the prevalence of diabetes was 11.3% among the poorest individuals and 4.3% among the wealthiest (difference of seven percentage points, 95% CI: 4.89 to 9.13, *p* < 0.001). There was no gap in the prevalence of diabetes across the poorest and wealthiest women in 2018 (13.9% vs. 12.2%, respectively, difference of 1.7 percentage points, 95% CI: −0.09 to 4.43, *p* = 0.209). Over the 13 years, the prevalence of diabetes in the poorest and wealthiest individuals increased 2.6 percentage points (95% CI: 0.02 to 4.99, *p* = 0.027) and 7.9 percentage points (95% CI: 5.42 to 10.44, *p* < 0.001), respectively.

**Figure 3:**
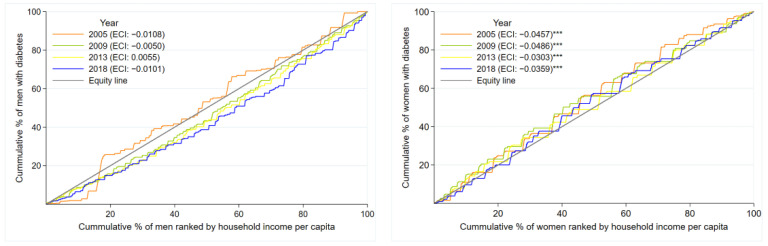
Concentration curves and the Erreygers concentration indices for diabetes prevalence according to sex. ECI: Erreygers concentration index. Test to compare 2005 ECI vs 2018 ECI: *p* = 0.9681 for men and *p* = 0.4754 for women. Significance level for ECI: *** = 0.01.

In the inequalities analysis, the ECIs for the general urban Argentinian population were −0.0304 (2005), −0.0286 (2009), −0.0141 (2013), and −0.0251 (2018). These results indicate that diabetes’ prevalence among the urban Argentinian population was concentrated among the poorest individuals. There was no difference in the ECIs values calculated for 2005 and 2018, indicating that in the study period, there was no progression in the reduction of inequalities for diabetes’ prevalence (which remained concentrated in the poorest). Figure 3 presents the CC and the ECIs for diabetes’ prevalence according to sex. For men, in 2005, the CC were above the line of equity, showing that the prevalence of diabetes was higher among the poorer. For the rest of the years, CC fell below the line of equity, indicating that the prevalence of diabetes was higher among the wealthiest subjects. However, the ECIs did not confirm these findings because they were not different from zero (*p* > 0.05 for all the years (2005, 2009, 2013, and 2018)). When we compared the ECI in 2005 versus 2018, there was no difference (*p* = 0.968). On the other hand, CCs for women over the 13 years were above the line of equity, showing that the prevalence of diabetes was higher among the poorer individuals. The ECIs over all the years were negative and different from zero at 1% of the significance level. When comparing the ECIs in 2005 and 2018, we did not find evidence reducing inequalities (*p* = 0.475).

Table 2 presents the decomposition of the inequality of diabetes’ prevalence in men and women during 2005 and 2018. For the elasticity findings, for men in 2005, the value of elasticity for being currently employed was −0.10, indicating that a 1% of change of individuals from currently unemployed to employed results in a 10% probability of a reduction in socioeconomic inequality in diabetes’ prevalence. For 2018, the reduction in socioeconomic inequality of a 1% of change of individuals from currently unemployed to currently employed was 9%. In women, the responsiveness of diabetes’ prevalence to a change in the variable currently employed was lower in comparison with men. For example, in 2005, an increase of 1% in individuals employed resulted in a reduction of 2.9% in socioeconomic inequality of diabetes’ prevalence. In 2018, the reduction of socioeconomic inequality driven by an increase of 1% of individuals employed was 4.7%. For the concentration indices, we found that during 2005 and 2018, the prevalence of diabetes for both women and men with primary or no education and with public insurance was more likely to be concentrated among the poorest individuals. In contrast, when comparing individuals who were married or cohabiting and were employed and individuals who had private or Social Security insurance, the prevalence of diabetes was more likely to be concentrated among the wealthiest individuals (Table 2).

Lastly, for the contribution and percentage contributions of the variables to the inequality in the prevalence of diabetes, our study found that during 2005 and 2018, socioeconomic inequality in men was largely driven by primary education, employment, and public insurance. On the other hand, Social Security or private insurance, marriage, and being between 30 and 59 years of age were contributors to the reduction in socioeconomic inequality. For women, during 2005, we found that socioeconomic inequality was largely driven by public insurance, primary education, employment, or being 60 years of age or older. During 2018, the key contributors of inequality were primary and secondary education, employment, and Social Security insurance. In 2005, the main contributor for a reduction in inequality was having Social Security insurance, while during 2018, the main contributor was public insurance and being between 30 and 59 years of age.

## 4. Discussion

Our study sought to measure the socioeconomic inequalities in the prevalence of diabetes in the adult population of Argentina’s urban areas. In summary, an increase in the prevalence of diabetes was found in the general urban population as well as in men and women during the 13-year study period (2005–2018). Likewise, women had a higher prevalence of diabetes than their male counterparts in the different years evaluated. No socioeconomic inequalities were reported in the prevalence of diabetes in men. In women and in the general population, a higher concentration of diabetes’ prevalence was found in lower socioeconomic groups, and no evidence of a reduction in inequalities was found during the study period.

An increase in the prevalence of diabetes was identified in Argentina’s urban population between 2005 and 2018, both in the general population as well as in men and women. This result is aligned with a previous report by the World Health Organization that described an increase in the prevalence of this disease in recent years to a value of around 10% of the adult Argentine population [28]. These results can be attributed either to the increase in life expectancy and the establishment of diabetogenic lifestyle patterns [29,30] or to the patterns of poor diet quality that have been reported previously in Argentina [31]. In the LAC region, including Argentina, the urban population has a higher prevalence of diabetes compared with that of rural areas [7,32,33]. This sustained increase in the prevalence of diabetes in urban areas indicates an increase in the burden of disease due to this health problem, as well as an increase in other complications and diseases, such as anxiety disorders [34].

In relation to sex, women were found to have a prevalence of diabetes greater than 10% in 2009, 2013, and 2018 (in men, the prevalence was greater than 10% only in 2018 [11.70%]). This population distribution pattern of diabetes prevalence in Argentina was previously reported [28] and is consistent with the worldwide prevalence patterns between sexes [35,36]. In Argentina, diabetes represents a significant burden of disease with approximately 1.3 million disability-adjusted life years (85% due to disabilities), with women having the highest burden of disease due to this cause (51% of years of lost life and 61% of years of life lived with disability, regardless of age) [37]. In addition, the literature describes that mortality is higher in women with diabetes compared with men, including a potential loss of the protective effect of sex for cardiovascular mortality [36,38]. Taking this into account, Argentine women represent a vulnerable population subgroup that requires special attention to the problem of diabetes since the burden of this disease seems to cause greater complications in this group.

A higher concentration of diabetes cases was found in the poorest population for all the years studied for both the general population and women. The influence of socioeconomic status on the presence of diabetes may vary according to the population evaluated. Studies in countries such as Bangladesh describe that the wealthiest population presents the highest prevalence of diabetes [39,40] in contrast with the result found for the urban population of Argentina. Studies conducted in LAC countries such as Chile report inequalities in the presence of diabetes, describing a greater number of cases among the poorest populations [41]. A study using the ENFR database reported that the higher the income was, the lower the prevalence was of unhealthy eating and poor health [42]. Since the influence of socioeconomic factors on the development of diabetes and of risk factors that may predispose a person to the development of this disease or other chronic diseases may differ according to the characteristics of the environment and the population studied, the study of socioeconomic inequalities is relevant for the identification of groups with greater vulnerability and for the development of strategies to mitigate the effect on the socioeconomic level of the prevalence of chronic diseases.

No reduction in inequality in the prevalence of diabetes was identified during the study period. In relation to this, in LAC countries, including Argentina, higher burdens of diabetes are described in the poorest regions [43,44]. The presence of inequalities is aligned with the differences in the prevalence of diabetes reported between cities in Argentina [45] and differences in mortality from diabetes in the geographic regions of Argentina [46]. In this sense, an analysis of socioeconomic inequalities in the prevalence of diabetes has implications for health decision-making, because it allows the identification of vulnerable groups and regions. Thus, it is necessary to allocate resources and design interventions for people of the lowest socioeconomic status. Some examples of interventions that can reduce the incidence of diabetes in vulnerable populations are increasing access to a healthy diet [47], increasing access and use of recreational spaces that promote physical activity [48], and the implementation of strategies to reduce the consumption of sugary drinks [49].

This study has some limitations that should be noted. First, ENFR was not designed to measure inequalities in the prevalence of diabetes. Second, the main outcome of the study was measured by self-reporting, which may lead to a lack of precision in the variables or recall bias. However, it has been described that self-reporting of diabetes has shown a high level of sensitivity compared with clinical diagnosis [50]. Third, the study design does not allow a causal relationship to be established between socioeconomic status and the presence of diabetes, and thus, long-term follow-up studies are needed to determine a causal relationship. Despite these limitations, we consider that using information from the only representative survey periodically carried out to measure health status in Argentina is useful for obtaining results of the state of diabetes’ prevalence in Argentina and for enabling the planning and administration of programs for the control of this chronic disease in urban areas.

## 5. Conclusions

The prevalence of diabetes increased during the 13 years of the study, with women having the highest prevalence. Differences were reported in the presence of diabetes according to socioeconomic status, and the majority of cases of this disease were concentrated in the population with a lower socioeconomic status. Given that no changes were observed in inequality, new strategies that favor healthy lifestyles and adequate control of the disease in this subgroup of the population are a must.

## Figures and Tables

**Table 1:** Sociodemographic characteristics of the study sample. ENFR 2005, 2009, 2013, and 2018.

	2005 (*n* = 41,219)	2009 (*n* = 34,583)	2013 (*n* = 32,232)	2018 (*n* = 29,094)
Variables	% (95% CI)	% (95% CI)	% (95% CI)	% (95% CI)
Sex				
Men	47.4 (46.25–48.46)	46.6 (45.77–47.48)	47.5 (46.39–48.53)	47.6 (46.53–48.58)
Women	52.6 (51.54–53.75)	53.4 (52.52–54.23)	52.5 (51.47–53.61)	52.4 (51.42–53.47)
Age in years				
Mean (standard deviation)	43.3 (17.94)	43.6 (17.99)	43.3 (17.87)	43.9 (17.77)
Married or cohabiting				
Yes	60.6 (60.56–60.60)	59.1 (59.03–59.07)	58.2 (58.13–58.17)	56.8 (56.74–56.78)
No	39.4 (39.40–39.44)	40.9 (40.93–40.97)	41.8 (41.83–41.87)	43.2 (43.22–43.26)
Education				
None	1.8 (1.83–1.84)	1.5 (1.53–1.54)	1.3 (1.30–1.31)	0.9 (0.96–0.97)
Primary	37.2 (37.20–37.24)	31.9 (31.84–31.88)	30.1 (30.09–30.12)	24.1 (24.13–24.16)
Secondary	36.9 (36.84–36.88)	39.7 (39.67–39.71)	41.2 (41.23–41.26)	43.1 (43.06–43.10)
Higher	24.1 (24.06–24.10)	26.9 (26.90–26.93)	27.4 (27.33–27.37)	31.8 (31.79–31.83)
Type of health insurance ^a^				
Private insurance	15.3 (15.30–15.33)	14.9 (14.89–14.92)	13.9 (13.92–13.94)	15.7 (15.71–15.73)
Social Security insurance	47.9 (47.89–47.94)	58.9 (58.87–58.91)	57.0 (56.94–56.98)	52.3 (52.24–52.28)
Public insurance	36.8 (36.75–36.79)	26.2 (26.18–26.22)	29.1 (29.09–29.13)	32.0 (32.00–32.04)
Currently employed?				
Yes	62.7 (62.65–62.69)	62.9 (62.87–62.91)	62.7 (62.72–62.76)	61.7 (61.64–61.67)
No	37.3 (37.31–37.35)	37.1 (37.09–37.13)	37.3 (37.24–37.28)	38.3 (38.33–38.36)
Household income per capita quintile ^b^				
Q1 (Poorest)	20.3 (20.31–20.34)	20.4 (20.45–20.48)	20.0 (20.02–20.05)	20.3 (20.28–20.31)
Q2	20.2 (20.19–20.22)	19.7 (19.64–19.67)	20.7 (20.73–20.76)	21.4 (21.34–21.38)
Q3	19.9 (19.95–19.98)	20.0 (19.98–20.01)	19.2 (19.22–19.25)	19.4 (19.35–19.38)
Q4	20.7 (20.70–20.73)	19.9 (19.85–19.88)	20.0 (20.04–20.07)	19.8 (19.79–19.82)
Q5 (Wealthiest)	18.8 (18.74–18.78)	20.0 (19.98–20.01)	19.9 (19.89–19.92)	19.1 (19.13–19.16)
Geographical region				
Metropolitan	39.3 (39.26–39.30)	36.3 (36.32–36.36)	37.4 (37.39–37.43)	38.8 (38.78–38.82)
Pampeana	33.2 (33.13–33.17)	35.1 (35.07–35.11)	33.4 (33.34–33.37)	30.9 (30.85–30.89)
Northwest	9.9 (9.88–9.90)	10.4 (10.36–10.38)	10.3 (10.24–10.26)	10.7 (10.70–10.73)
Northeast	6.8 (6.83–6.85)	7.2 (7.23–7.25)	7.3 (7.32–7.34)	7.7 (7.66–7.68)
Cuyo	6.4 (6.41–6.43)	6.5 (6.44–6.46)	6.5 (6.44–6.46)	6.5 (6.51–6.53)
Patagonia	4.4 (4.41–4.43)	4.5 (4.51–4.53)	5.2 (5.20–5.22)	5.4 (5.42–5.43)

All estimates included the weighting factor and sample specifications for each survey. ENFR: “National Survey of Risk Factors” (for its acronym in Spanish). ^a^ The sample size by year was 40,897, 32,832, 32,063, and 29,070 for 2005, 2009, 2013, and 2018, respectively. ^b^ The household income per capita was estimated by the consumer unit following the OECD criteria.

**Table 2:** Decomposition of Erreygers concentration index.

	2005 Men				2018 Men				2005 Women				2018 Women			
Variable	Elasticity	CI	Contribution	% Contribution	Elasticity	CI	Contribution	% Contribution	Elasticity	CI	Contribution	% Contribution	Elasticity	CI	Contribution	% Contribution
Age group, in years																
18–29	Base	Base	Base	Base	Base	Base	Base	Base	Base	Base	Base	Base	Base	Base	Base	Base
30–59	0.1691	0.0749	0.0127	−117.7130	0.2139	0.1165	0.0249	−246.3620	0.1298	0.1321	0.0172	−37.5261	0.1036	0.1424	0.0148	−41.1064
60 or more	0.1128	–0.0561	−0.0063	51.8197	0.1455	–0.0473	−0.0069	68.0948	0.1085	–0.0948	−0.0103	22.4872	0.0775	–0.0626	−0.0049	13.5258
Married or cohabiting																
Yes	0.0205	0.0233	0.0005	−4.2448	0.0575	0.1159	0.0067	−65.8559	0.0343	0.1665	0.0057	−12.4906	0.0379	0.1845	0.0070	−19.4643
No	Base	Base	Base	Base	Base	Base	Base	Base	Base	Base	Base	Base	Base	Base	Base	Base
Education																
None	0.0010	−0.0318	0.0000	0.2962	−0.0019	–0.0118	0.0000	−0.2222	0.0040	–0.0360	−0.0001	0.3157	0.0036	–0.0144	−0.0001	0.1460
Primary	0.0468	−0.3678	−0.0172	159.9779	0.0042	−0.2448	−0.0010	10.2248	0.0377	–0.3769	−0.0142	31.1138	0.0673	–0.2245	−0.0151	42.0503
Secondary	0.0235	0.0122	0.0003	−2.6606	−0.0079	−0.0729	0.0006	−5.7288	0.0048	0.0313	0.0002	−0.3315	0.0504	–0.1145	−0.0058	16.0739
Higher	Base	Base	Base	Base	Base	Base	Base	Base	Base	Base	Base	Base	Base	Base	Base	Base
Type of health insurance																
Private insurance	0.0098	0.1892	0.0019	−17.2940	0.0282	0.1892	0.0053	−52.7814	0.2111	0.0211	0.0042	−9.2671	-0.0181	0.2137	−0.0039	10.7629
Social Security insurance	0.0364	0.2851	0.0104	−96.5498	0.1214	0.1916	0.0233	−230.0361	0.0860	0.2552	0.0219	−48.0006	−0.0599	0.1434	−0.0086	23.9317
Public insurance	0.0121	−0.4625	−0.0056	52.1129	0.0571	−0.3804	−0.0217	214.6260	0.0786	−0.4621	−0.0363	79.4977	–0.0387	−0.3502	0.0136	−37.7785
Currently employed?																
Yes	−0.1010	0.1069	−0.0108	100.3942	−0.0974	0.1522	−0.0148	146.5333	−0.0299	0.1658	−0.0050	10.8341	−0.0476	0.2039	−0.0097	27.0251
No	Base	Base	Base	Base	Base	Base	Base	Base	Base	Base	Base	Base	Base	Base	Base	Base
Household income per capita quintile ^a^																
Q1 (Poorest)	0.0010	−0.5309	−0.0005	4.7730	0.0166	–0.5794	−0.0096	95.3637	0.0275	−0.6709	−0.0269	57.7319	−0.0094	−0.6385	0.0060	−16.7285
Q2	0.0002	−0.3371	−0.0001	0.5097	0.0063	−0.2979	−0.0019	18.5930	0.0331	−0.2525	−0.0083	18.2575	0.0079	−0.2543	−0.0020	5.5669
Q3	−0.0191	−0.0581	0.0011	−10.3427	–0.0018	−0.0125	0.0000	−0.2262	0.0312	0.0455	0.0014	−3.1105	0.0110	0.0199	0.0002	−0.6104
Q4	0.0040	0.2888	0.0012	−10.8355	−0.0020	0.2713	−0.0005	5.3929	0.0362	0.3462	0.0125	−27.4487	–0.0074	0.3043	−0.0022	6.2332
Q5 (Wealthiest)	Base	Base	Base	Base	Base	Base	Base	Base	Base	Base	Base	Base	Base	Base	Base	Base
Explained inequality			−0.0126	110.2433			0.0043	−42.3842			−0.0380	82.0629			−0.0106	29.6277
Residual			−0.0019	−10.2433			0.0144	142.3842			0.0077	17.9371			0.0253	70.3723

^a^ The household income per capita is adjusted by the consumer unit following the OECD criteria. Abbreviation. CI, concentration index.

## Data Availability

Publicly available datasets were analyzed in this study. This data can be found here: https://www.indec.gob.ar/ (accessed on 26 May 2022).
